# The Psychometric Properties of the Behavioural Regulation in Exercise Questionnaire (BREQ-3): Factorial Structure, Invariance and Validity in the Italian Context

**DOI:** 10.3390/ijerph19041937

**Published:** 2022-02-09

**Authors:** Elisa Cavicchiolo, Maurizio Sibilio, Fabio Lucidi, Mauro Cozzolino, Andrea Chirico, Laura Girelli, Sara Manganelli, Francesco Giancamilli, Federica Galli, Pierluigi Diotaiuti, Arnaldo Zelli, Luca Mallia, Tommaso Palombi, Dario Fegatelli, Flavia Albarello, Fabio Alivernini

**Affiliations:** 1 Department of Human, Philosophical and Educational Sciences, University of Salerno, 84084 Fisciano, Italy; msibilio@unisa.it (M.S.); mcozzolino@unisa.it (M.C.); lgirelli@unisa.it (L.G.); 2 Department of Developmental and Social Psychology, Sapienza University of Rome, 00185 Rome, Italy; fabio.lucidi@uniroma1.it (F.L.); andrea.chirico@uniroma1.it (A.C.); francesco.giancamilli@uniroma1.it (F.G.); tommaso.palombi@uniroma1.it (T.P.); dario.fegatelli@gmail.com (D.F.); flavia.albarello@uniroma1.it (F.A.); fabio.alivernini@uniroma1.it (F.A.); 3 National Institute for the Evaluation of the Education System (INVALSI), 00153 Rome, Italy; sara.manganelli@invalsi.it; 4 Department of Movement, Human and Health Sciences, University of Rome “Foro Italico”, 00185 Rome, Italy; federica.galli@uniroma1.it (F.G.); arnaldo.zelli@uniroma4.it (A.Z.); luca.mallia@uniroma4.it (L.M.); 5 Department of Human Sciences, Society and Health, University of Cassino and Southern Lazio, 03043 Cassino, Italy; p.diotaiuti@unicas.it

**Keywords:** Behavioral Regulation in Exercise Questionnaire, BREQ-3, Italian validation, motivation, self-determination theory, exercise

## Abstract

Background: Motivation to engage in physical activity plays a central role in ensuring the health of the population. The present study investigated the psychometric properties and validity in Italy of the Behavioral Regulation in Exercise Questionnaire (BREQ-3), a widely used instrument for assessing individuals’ motivation to exercise based on self-determination theory (SDT). Methods: A large sample (N = 2222; females = 55.4%; *M*_age_ = 36.4 years, *SD*_age_ = 13.9, min = 20, max = 69) of young people, and middle aged and older adults completed the Italian translation of the BREQ-3, also indicating their intentions to exercise in the following weeks. Results: Confirmatory factor analyses showed that the posited six-factor structure of the BREQ-3 fitted the data well (CFI = 0.96; RMSEA = 0.05; SRMR = 0.04) and provided evidence for full measurement invariance across gender and different age groups. The construct validity of the BREQ-3 was supported by the latent correlations among the subscales, which were consistent with the quasi-simplex pattern theorized by SDT. The overall level of self-determination and the intention to exercise were positively correlated, providing evidence for the criterion validity of the scale. Conclusions: The Italian version of the BREQ-3 has proved to be a reliable and valid instrument for measuring the behavioral regulation of exercise in individuals with different demographic characteristics.

## 1. Introduction

The positive effects of exercise on physical and mental health are well known [1], but there is substantial evidence that in industrialized countries most adults are insufficiently active [2,3,4]. This issue is of great concern in the context of public health initiatives. Many people are not adequately motivated to engage in and sustain a physically active lifestyle [5]. Self-determination theory (SDT) [6,7] has been widely used to study motivational processes in diverse contexts such as education [8,9,10,11,12,13,14], sports [15,16,17], and health [18,19,20] and it has received increasing attention also in the domain of exercise [21,22,23,24,25]. According to SDT, human motivation has a multidimensional structure based on different types of motivation (or regulatory styles) that reflect various levels of self-determination (the perception of being the source of one’s own behavior). SDT identifies two main forms of motivation: intrinsic motivation, when people engage in activities due to their inherent interest and joy [26] and extrinsic motivation referring to activities that are carried out for reasons other than their inherent satisfactions [12]. Intrinsic motivation is the most self-determined form of behavior, and individuals who are intrinsically motivated to exercise do so simply since they enjoy it (perhaps stating: “I like my daily walk and I feel good when I’m doing it”) rather than for some instrumental reason. Being intrinsically motivated leads individuals to experience pleasant emotions and to feel free and relaxed [27]. Several studies have shown that intrinsic motivation is a crucial factor in learning over an individual’s entire lifespan [7] and that it has positive effects on well-being and life satisfaction, persistence, participation in activities and high levels of performance [5,19,28,29,30,31,32].

SDT specifies four different forms of extrinsic motivation, according to their relative degree of autonomy, which range from controlled forms of regulation (external and introjected regulation) to autonomous enacted regulation (identified and integrated regulation). External regulation is a controlled type of motivation, which concerns behaviors that are performed in order to satisfy an external demand or that are driven by externally imposed rewards and punishments [6]. An example of this might be exercising since our doctor or family members tell us we should. Introjected regulation is an extrinsic motivation that has been partially internalized [12], in such a way that behavior is regulated by internal rewards in the form of self-esteem and the avoidance of anxiety, shame, or guilt [12]. People with this form of motivation exercise since otherwise they would feel bad or guilty if, for example, they miss their weekly training. Identified regulation is an autonomous enacted form of motivation, in which a personal importance and value is attributed to the behavior, thereby increasing the volition to act, even if the activity in itself is seen as unpleasant. In this case individuals exercise since they can see the future benefits for them in doing so, or due to the fact that they think that it is important for them. Integrated regulation is the most autonomous form of extrinsic motivation and it occurs when identified forms of regulation are assimilated to the self [33] and the behavior is congruent with the individual’s other interests and values. Integrated regulation is different from intrinsic motivation since it is still instrumental (even though it is based on a sense of value) rather than being pursued for interest or enjoyment [26]. Individuals who are motivated by integrated regulation exercise since they consider it as part of their identity and coherent with their aims in life. SDT also refers to amotivation, which concerns lacking intentionality [12,34]. Individuals who lack motivation (whether intrinsic or extrinsic) do not perceive any contingency between their actions and the relative outcomes and are unable to find any good reason to perform an activity [27,35]. They are therefore very unlikely to continue with exercise or training that they may have begun.

All of the different forms of motivation and amotivation specified above can be placed on a self-determination continuum that reflects their relative degree of autonomy. The structure of the self-determination continuum according to SDT [36] implies that there is a quasi-simplex (ordered) pattern of correlations between the different forms of motivation, with stronger positive correlations between those that are adjacent (e.g., intrinsic motivation and integrated regulation) than those that are not (e.g., intrinsic motivation and external regulation). Several studies have provided support for this simplex pattern of correlations [37,38,39,40,41,42,43,44], and also in the domain of exercise [45,46,47,48].

Over the years, several instruments have been developed to measure human motivation according to SDT in various different domains. The original Behavioral Regulation in Exercise Questionnaire (BREQ), developed by Mullan et al. [49], was the first attempt to measure different types of regulation in the domain of exercise as a multidimensional construct. The BREQ assesses external, introjected and identified regulation, as well as intrinsic motivation, and several studies have provided support for its validity and reliability [50,51,52,53]. However, this first version didn’t include the category of amotivation and a new revised version of the instrument was therefore developed. The BREQ-2 [54] consists of 19 items, including four subscales for assessing the various forms of motivation (external, introjected, identified and intrinsic), with the addition of four items for measuring amotivation. Respondents reply to the question “Why do you engage in exercise?” on a scale ranging from 0 (“not true for me”) to 4 (“very true for me”). The BREQ-2 has become one of the most widely used instruments in the exercise domain and several studies conducted in different countries have provided support for its validity and reliability [27,55,56,57,58,59]. In Italy, the BREQ-2 was validated by Costa and colleagues [57] on a sample of 576 gym users. Internal reliability, construct validity and criterion validity were assessed, and the factorial structure of the scale was confirmed by means of an exploratory factor analysis. Overall, the results confirmed the good psychometric characteristics of the BREQ-2, indicating that it is a useful instrument for assessing motivation in the domain of exercise on the basis of SDT, also in the Italian context.

Unfortunately, the BREQ-2 did not include integrated regulation, and yet another version of the instrument, the BREQ-3 [48,54], was proposed. The inclusion of the subscale of integrated regulation has made it possible to better understand the different motivational processes at work in the sphere of physical exercise and, as pointed out by Cid and colleagues [46] (p. 2), it helps us to understand “the gap between accepting the behavior and obtaining a separable and pleasurable outcome”. The BREQ-3 contains 24 items, 4 for each subscale and it has been applied extensively to the domain of exercise [5]. The BREQ-3 has several features. For example, it assesses the whole continuum of motivation according to SDT and it is a short scale that is easy to administer in most circumstances. However, while the studies based on the BREQ-2 seem to highly support its reliability and validity (e.g., [27]), at present we have little evidence regarding the psychometric properties of the BREQ-3, especially across different cultural contexts. Wilson and colleagues [48] were the first to include four items for measuring integrated regulation within the BREQ. They examined the factor structure of the scale, firstly in a sample of undergraduate psychology students (N = 207; 29.5% males; *M*_age_ = 19) enrolled in a Canadian university, and secondly in a sample of exercisers enrolled in a running club in central Canada (N = 132; 95.3% female; *M*_age_ = 47.5 years). In both these studies, the scale consisted of 19 items and a 5-factor model was tested, but no items for amotivation were included. The results provided evidence for the validity of this new version of the instrument containing the integrated regulation items. The psychometric properties of the scale were then examined in a sample of adults in Brazil (N = 1041; 55% male; age > 18) and in this study the results of the confirmatory factor analysis (CFA) corroborated the 6-factor structure of a 23-item version of the scale and indicated its measurement invariance across gender and age [60]. In the European context, at present a Spanish and a Portuguese version of the BREQ-3 are available [46,47,61]. A Spanish study examined the psychometric properties of a 6-factor scale with 23 items in a sample of young people and adults (N = 524; 51.5% male; *M*_age_ = 29.6). The results of the CFA revealed acceptable fit indices; the diverse subscales supported the simplex pattern; and the factor structure was invariant across gender and age [47]. In Portugal, two studies investigated the factor structure and psychometric properties of the BREQ-3. The first study was conducted on two independent samples of Portuguese exercisers (N = 448 calibration; 374 validation; 60.2% female; *M*_age_ = 40.3). The results showed that the 6-factor model with 24 items did not have a satisfactory fit to the data. After removing six items (one for each factor), the shorter 18-item version substantially improved the fit of the model in both samples (calibration and validation). The results also showed model invariance across gender [46]. The second study involved a sample of students, teachers, and staff of a tertiary education polytechnic (N = 996; 82.6% female, *M*_age_ = 23.4). The results of the CFA provided empirical support for a 6-factor structure (with 23 items) of the BREQ-3 in the Portuguese context [61].

The present study was intended to remedy the fact that no research had as yet been conducted in the Italian context. We analyzed the factorial structure, validity, and reliability of the BREQ-3 based on a large sample (N = 2222) of Italian young people and adults. We adopted the recently developed 18-item version of the BREQ-3 since it is more time-efficient and does not include six items of the original version which have proved to be problematic.

## 2. Aims and Objectives

The main objective of the present study is to establish the psychometric properties and the validity of the 18-item version of the BREQ-3 in the Italian context. In order to achieve the goal of the study and on the basis of the literature summarized above, we tested the following hypotheses. Firstly, we assumed the six-factor structure of the BREQ-3, as posited by SDT. Secondly, we hypothesized that the measure was invariant across gender and age groups. At the present time there is a lack of studies that consider the measurement invariance across very different age groups. Thirdly we would expect to detect the quasi-simplex pattern posited by SDT, with stronger positive correlations between adjacent subscales than between subscales that are further apart. This would provide evidence for the construct validity of the BREQ-3. Finally, we would expect to find a non-trivial positive correlation (*r* > 0.30) between the relative autonomy index (RAI) [36,62] and the intention to exercise. This would support the criterion validity of the scale.

## 3. Materials and Methods

### 3.1. Sample and Procedure

The participants in the present study were 2222 young people and adults from several different geographical areas of Italy. A group of Italian researchers coordinated the study, which consisted of an online survey about the habits and behavior of young people and adults with respect to exercise. Information was collected about their different intentions and motives for doing exercise as well as their demographic characteristics. The study was conducted in 2021 with the use of the online software “Alchemer” specifically designed for online surveys (https://www.alchemer.com. (accessed on 6 December 2021)). The participants were recruited using online advertisements. After giving their initial consensus, 12 participants then decided not to take part in the research (response rate 99.5%).

The average age of participants was 36.4 years (*SD* = 13.9; min = 20, max = 69), with a slight prevalence of females (55.4%).

All of the participants were informed regards the general purpose of the study and their rights to anonymity and confidentiality. All of the participants who took part in the study provided their informed written consent. It took approximately 10 min to complete the survey. The study protocol was approved by the Ethics Committee of the Sapienza University of Rome. The dataset analyzed during this study are not publicly available, but are available from the corresponding author on reasonable request.

### 3.2. Measures

The Behavioral Regulation Exercise Scale (BREQ-3) [48,54] consists of six subscales assessing amotivation, external, introjected, identified, integrated regulation and intrinsic motivation. The BREQ-2, a previous version of the instrument that did not include integrated regulation, has been validated in the Italian context [57], but before we conducted our study an analysis of the structure and the validity of the BREQ-3 scale in Italy was lacking. In the present study, we used the recently developed 18-item version of the instrument [46]. The BREQ-3 was translated from English into Italian by the authors and then back-translated by a person fluent in English and Italian. A team of independent judges then considered the equivalence of the original and the back-translated versions of the scales. After discussing instances of non-equivalence, the final version was drawn up.

The items of the Italian version of the BREQ-3 are reported in Appendix A. Responses were recorded on a 5-point scale ranging from 0 (“not true for me”) to 4 (“very true for me”) and the relative autonomy index (RAI) was calculated. The RAI is a scoring method in which each of the regulation subscales is calculated before being weighted and combined with other forms of regulation, in accordance with their expected position on the SDT continuum [63]. This results in a single score representing the degree of relative autonomy. The RAI therefore indicates a person’s overall motivational orientation in such a way that lower levels of autonomous motivation are indicated by lower negative RAI scores, while higher levels of autonomous motivation are indicated by higher positive RAI scores. In the present study, the RAI scores ranged from −19 to +24.

Intention to exercise in the following weeks was measured by a single item (“How likely are you to exercise in the next two months?”). This item was adapted from previous studies conducted in Italy [64,65]. The responses were recorded on a 7-point scale ranging from 1 (“not at all likely”) to 7 (“very likely”).

Gender was coded into two categories, with 1 indicating males and 2 indicating females. Age was grouped into the three categories of young adulthood (18 to 35 years), middle age (36 to 55 years), and older adulthood (over 55 years) as had been carried out in previous studies (e.g., [66]).

### 3.3. Analysis

The posited model with six correlated factors was estimated by using the Mplus 8 software, version 1.6 [67]. Goodness-of-fit of the model with the data was assessed using the maximum likelihood robust (MLR) chi-square test statistic and multiple fit indices (comparative fit index [CFI], root mean square error of approximation [RMSEA], and standardized root mean square residual [SRMR]), according to the cut-off values for well-fitting models [68]. The measurement invariance of the scale across gender and age groups was examined by means of a hierarchical series of multigroup confirmatory factor analyses (CFAs), imposing increasingly restrictive equality constraints on the model’s parameters in accordance with the indications of Van de Schoot, Lugtig, and Hox [69]. In each step of the analysis the fit of the nested models was compared using the change in CFI values (ΔCFI ≤ 0.01) according to Cheung and Rensvold [70]. In order to support the construct validity of the instrument, the latent correlations between the six subscales were analyzed. Finally, we calculated the RAI scores, in order to investigate the hypothesized relationships between the BREQ-3 and a criterion-related variable. The formula we have used was: (amotivation x (−3)) + (external regulation x (−2)) + (introjected regulation x (−1)) + (identified regulation x (+1)) + (integrated regulation x (+2)) + (intrinsic motivation x (+3)) [54,71]. The Pearson’s correlation was computed between the RAI scores and the intention to exercise.

Missing data were not present in our database since the online survey was set up with mandatory answers to all questions.

## 4. Results

### 4.1. Descriptive Statistics and Correlations

Table 1 shows bivariate correlations between the items of the BREQ-3 scale. The means, standard deviations, and McDonald’s omega for each subscale are also reported in Table 1.

### 4.2. The Factorial Structure of the BREQ-3

**Figure 1:**
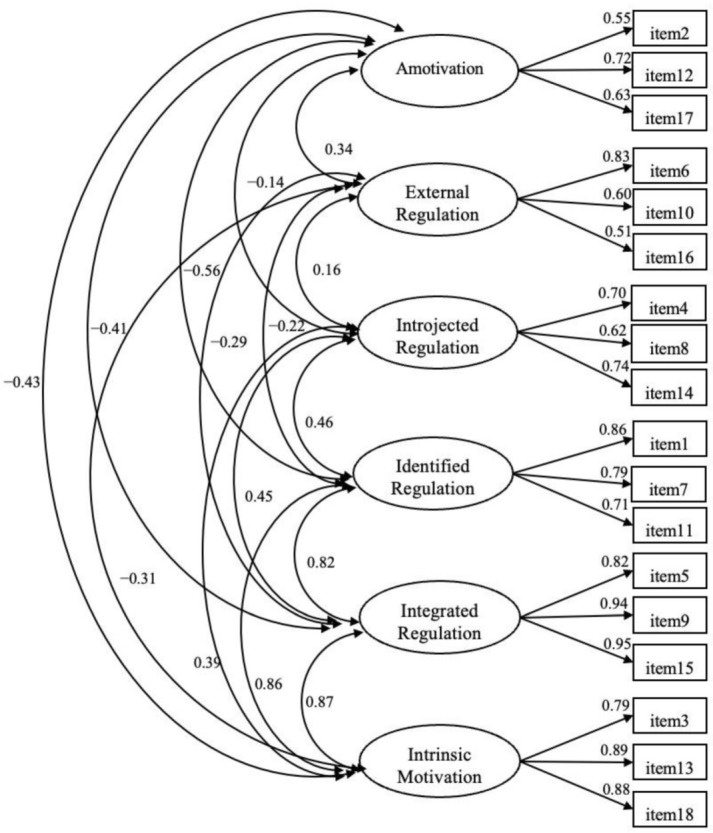
Confirmatory factor analysis results. Note: All of the estimates are standardized. All of the estimates are statistically significant *p* < 0.001.

The results of the confirmatory factor analysis (CFA) confirm the posited six-factor structure for the BREQ-3: χ2 (120) = 833.987, *p* < 0.001; CFI= 0.96; RMSEA = 0.05; SRMR = 0.04. Except for the chi-square test (probably affected by the large size of the sample we used in the present study), all of the fit indices indicated a good fit of the model with the empirical data [68,72]. In Table 2 the goodness-of-fit indices of the BREQ-3 model for the present study are reported, along with the results for other existing versions. The standardized factor loadings are presented in Figure 1. All of the loadings were statistically significant (*p* < 0.001) and ranged from 0.51 to 0.95.

### 4.3. Measurement Invariance across Gender and Age Groups

The results of the multigroup CFAs across gender and age groups are presented in Table 3. In the multigroup CFAs across gender, the comparison of the configural invariance model with the model with all of the factor loadings constrained to be equal across groups (metric invariance model) showed that the difference in the CFI between the models was smaller than the cut-off criterion (ΔCFI = −0.001) providing support for the hypothesis of metric invariance of the scales across gender. The results of the comparison of the metric invariance model with the model in which all of the item intercepts were constrained to be equal across groups (scalar invariance model) provided support for the full scalar invariance of the scales (ΔCFI = 0.001). In the multigroup CFAs across age groups, the comparison of the configural invariance model with the metric invariance model confirmed the metric invariance of the scales (ΔCFI = 0.003). Finally, the comparison of the metric invariance model with the scalar invariance model showed the full scalar invariance of the scales (ΔCFI = 0.008). The overall results provided support for the full scalar invariance of the scales across gender and age groups.

### 4.4. Latent Correlations among the BREQ-3 Subscales

The quasi-simplex pattern posited by SDT (see Figure 1 and Table 4), with stronger positive correlations between adjacent subscales than between subscales that are further apart was substantially supported by the data. This result provided evidence for the construct validity of the BREQ-3.

### 4.5. Relationship between the Overall Level of Self-Determination (RAI) and the Intention to Exercise

The Pearson’s correlation between the RAI scores and the intention to exercise showed that they were positively correlated, with *r* = 0.55 (*p* < 0.001). This result supported the criterion validity of the BREQ-3.

## 5. Discussion

The present study aimed to investigate the factorial structure, invariance, and validity of the BREQ-3 in a large sample of young people and middle aged and older adults in the Italian population.

As regards the factorial structure of the instrument, the posited measurement model had a good fit to the data, showing six related but distinct factors: intrinsic motivation, integrated, identified, introjected, and external regulation, and amotivation. This is consistent with validation studies conducted in other contexts such as Spain, Brazil, and Portugal [46,47,60,61].

All of the BREQ-3 subscales showed full measurement invariance across gender and different age groups. Males and females as well as younger and older adults therefore appear to interpret the corresponding items in a very similar way. These results extend the findings of previous studies [57], indicating that the BREQ-3 scores can be meaningfully used to compare the different types of motivation across groups of widely varying ages. The present study also provided evidence for the construct validity of the instrument, since our data showed that the correlation between forms of motivation that are adjacent on the continuum is stronger than the correlation between more distal types of motivation, thereby supporting the simplex pattern posited by SDT. This pattern of correlations is consistent with previous studies conducted in different domains [37,38,39,40,41,42,43,44,45,48] and it is also supported by the results of some validation studies of the BREQ-3 conducted in other cultural contexts [46,47]. This result is especially important since construct validity analyses are not frequently performed, which can lead to reporting biased results [73]. Finally, the criterion validity of the BREQ-3 was supported by a significant and non-trivial correlation between the individual’s overall level of self-determination (the RAI) and the intention to exercise. This result is in line with previous research showing a statistically significant correlation between autonomous forms of motivation and intention to exercise [22,45,51,74,75], and it is confirmed by another validation study of the BREQ-3 [48].

Understanding the different forms of motivation that individuals experience during exercise can help to forecast the possible outcomes [74], and we believe that the present study has made a significant contribution to the literature in several ways. Firstly, we have expanded the range of instruments available for motivational research by validating the BREQ-3 in the Italian context, thereby making it possible to conduct international comparisons. In addition, we have for the first time addressed the issue of the measurement invariance of the BREQ-3 across very different age groups, including older adults, providing evidence for its use to meaningfully compare motivational processes in the exercise domain.

Despite all of the strengths of the BREQ-3, some limitations of the present study should be mentioned. Although our data came from a large sample of young people and adults, only the Italian population was considered. Future research should therefore be conducted to generalize our findings across different countries and cultures. Moreover, we investigated the relationship between the RAI and the intention to exercise in a cross-sectional study. Finally, some further aspects of the validity of the BREQ-3 could be verified in future. For example, for the purpose of the present study, we included only motivational consequences while future research in the Italian context may benefit from an investigation of the relationships between this scale and the determinants of self-determination, in order to test a more complete model of people’s motivations for doing exercise.

## 6. Conclusions

The Italian version of the BREQ-3 has proved to be a concise, reliable, and versatile instrument that has broad applicability in different exercise contexts and that assesses all of the forms of behavioral regulation proposed by SDT. Moreover, we showed for the first time that the BREQ-3 is invariant across very different age groups, suggesting that during young adulthood, middle age, and old age individuals appear to conceptualize the various forms of motivation to exercise in the same way.

## Figures and Tables

**Table 1:** Zero-order correlations between the items of the scale, means, standard deviations, and McDonald’s omega of the BREQ-3 subscales.

	Mean	*SD*	ω	Correlations																	
				1	2	3	4	5	6	7	8	9	10	11	12	13	14	15	16	17	18
Amotivation	0.22	0.52	0.65																		
Item2				-																	
Item12				0.42 **	-																
Item17				0.30 **	0.46 **	-															
External	0.67	0.82	0.69																		
Item6				0.17 **	0.20 **	0.22 **	-														
Item10				0.08 **	0.09 **	0.11 **	0.49 **	-													
Item16				0.11 **	0.09 **	0.10 **	0.40 **	0.40 **	-												
Introjected	1.55	1.05	0.74																		
Item4				−0.10 **	−0.10 **	−0.10 **	0.08 **	0.07 **	0.03	-											
Item8				−0.02	−0.00	−0.00	0.16 **	0.06 **	0.04	0.42 **	-										
Item14				−0.05 *	−0.07 **	−0.07 **	0.08 **	0.05 **	0.04 *	0.51 **	0.49 **	-									
Identified	3.39	0.83	0.83																		
Item1				−0.27 **	−0.30 **	−0.34 **	−0.21 **	−0.11 **	−0.09 **	0.33 **	0.13 **	0.24 **	-								
Item7				−0.28 **	−0.36 **	−0.31 **	−0.12 **	−0.04	−0.06 **	0.33 **	0.16 **	0.24 **	0.69 **	-							
Item11				−0.19 **	−0.24 **	−0.25 **	−0.10 **	−0.04 *	−0.05 **	0.38 **	0.23 **	0.32 **	0.60 **	0.57 **	-						
Integrated	2.77	1.3	0.94																		
Item5				−0.23 **	−0.24 **	−0.29 **	−0.18 **	−0.08 **	−0.07 **	0.38 **	0.18 **	0.30 **	0.69 **	0.64 **	0.55 **	-					
Item9				−0.20 **	−0.23 **	−0.27 **	−0.27 **	−0.11 **	−0.09 **	0.34 **	0.19 **	0.31 **	0.67 **	0.56 **	0.57 **	0.77 **	-				
Item15				−0.22 **	−0.26 **	−0.29 **	−0.27 **	−0.11 **	−0.10 **	0.35 **	0.19 **	0.32 **	0.69 **	0.58 **	0.55 **	0.77 **	0.91 **	-			
Intrinsic	2.89	1.06	0.89																		
Item3				−0.16 **	−0.23 **	−0.23 **	−0.22 **	−0.09 **	−0.08 **	0.27 **	0.12 **	0.19 **	0.59 **	0.52 **	0.45 **	0.60 **	0.65 **	0.65 **	-		
Item13				−0.18 **	−0.22 **	−0.27 **	−0.26 **	−0.13 **	−0.11 **	0.28 **	0.13 **	0.24 **	0.64 **	0.57 **	0.54 **	0.65 **	0.71 **	0.73 **	0.74 **	-	
Item18				−0.23 **	−0.28 **	−0.31 **	−0.24 **	−0.12 **	−0.10 **	0.32 **	0.15 **	0.27 **	0.68 **	0.62 **	0.57 **	0.66 **	0.71 **	0.75 **	0.67 **	0.79 **	-

Note: ** *p* < 0.01; * *p* < 0.05. *SD*, standard deviation; ω, McDonald’s omega.

**Table 2:** Goodness-of-fit indices of the BREQ-3 model (including other existing versions).

	N	χ2	*df*	χ2/*df*	CFI	RMSEA
English version (BREQ-3) *	207	357.51	142	2.51	0.92	0.09
English version (BREQ-3) *	132	253.82	142	1.79	0.93	0.09
Brazilian version (BREQ-3) **	1041	406.35	215	1.89	0.93	0.07
Spanish version (BREQ-3) ***	524	689.13	215	3.21	0.91	0.06
Portuguese version (BREQ-3) ****	996	931.69	215	4.33	0.98	0.05
Portuguese version (BREQ-3) *****	374	254.08	120	2.11	0.95	0.06
Italian version (present study)	2222	833.987	120	6.94	0.96	0.05

Note: χ2 chi-squared; *df*, degrees of freedom; χ*/*df*, normative chi-square; CFI, comparative fit index; RMSEA, root mean square error of approximation; * [48]; ** [60]; *** [47]; **** [46]; ***** [61].

**Table 3:** Goodness-of-fit indices for invariance of the BREQ-3 across gender and age groups.

	χ2	df	χ2/df	CFI	RMSEA	SRMR	Models Compared	ΔCFI
Gender (male/female)								
Configural model	984.389	240	4.102	0.953	0.053	0.041		-
Metric model	981.368	252	3.894	0.954	0.051	0.042	Metric against configural	−0.001
Scalar model	1005.798	264	3.810	0.953	0.050	0.042	Scalar against metric	0.001
Age groups (18 to 35 years/36 to 55 years/over 55 years)								
Configural model	1107.438	360	3.076	0.954	0.053	0.041		-
Metric model	1177.256	384	3.065	0.951	0.053	0.047	Metric against configural	0.003
Scalar model	1341.339	408	3.287	0.943	0.056	0.050	Scalar against metric	0.008

**Table 4:** Latent correlations between the different subscales.

	Amotivation	External Regulation	Introjected Regulation	Identified Regulation	Integrated Regulation	Intrinsic Motivation
Amotivation	-					
External regulation	0.340 ***	-				
Introjected regulation	−0.137 ***	0.156 ***	-			
Identified regulation	−0.559 ***	−0.215 ***	0.462 ***	-		
Integrated regulation	−0.408 ***	−0.293 ***	0.449 ***	0.823 ***	-	
Intrinsic motivation	−0.429 ***	−0.308 ***	0.385 ***	0.855 ***	0.866 ***	-

Note: *** *p* < 0.001.

## Data Availability

Data are available under request to the corresponding author.

## Appendix A

The 18-item version of the Italian BREQ-3 scale. Amotivation: item 2, 12, 17. External Regulation: item 6, 10, 16. Introjected Regulation: item 4, 8, 14. Identified Regulation: item 1, 7, 11, Integrated Regulation: item 5, 9, 15. Intrinsic Motivation: 3, 13, 18.

The original version of the BREQ-3 is available at the following link: http://exercise-motivation.bangor.ac.uk/breq/breqdown.php (accessed on 20 September 2021).

INSTRUCTIONS. PERCHE’ FA ATTIVITA’ FISICA?

Vorremmo conoscere le ragioni che spingono le persone a fare o non fare attività fisica.

Le ricordiamo che non esistono risposte giuste o sbagliate, ma solo risposte che descrivono meglio ciò che lei pensa dell’attività fisica. Le sue risposte saranno confidenziali e verranno utilizzate solo per gli scopi della presente ricerca.

Per favore, risponda indicando in che misura le seguenti affermazioni sono vere nel suo caso, da 0 (“non è vero per me”) a 4 (“è molto vero per me”).

**BREQ-3 Items—Italian Version**
1. Fare attività fisica è importante per me
2. Non vedo il motivo per cui dovrei svolgere attività fisica
3. Svolgo attività fisica perché è divertente
4. Mi sento in colpa se non faccio attività fisica
5. Faccio attività fisica perché è coerente con i miei obiettivi di vita
6. Faccio attività fisica perché gli altri mi dicono che dovrei farlo
7. Perché riconosco i benefici di svolgere attività fisica
8. Mi vergogno quando salto una sessione di attività fisica
9. Considero l’attività fisica parte della mia identità
10. Svolgo attività fisica perché i miei amici/familiari/partner dicono che dovrei
11. Credo che sia importante fare lo sforzo di fare attività fisica regolarmente
12. Non vedo la ragione per svolgere attività fisica
13. Ritengo che fare attività fisica sia molto piacevole
14. Mi sento un fallimento quando non faccio attività fisica per un po’
15. Considero l’attività fisica una parte fondamentale di me
16. Svolgo attività fisica perché gli altri non sarebbero contenti di me se non lo facessi
17. Penso che svolgere attività fisica sia una perdita di tempo
18. Traggo piacere e soddisfazione dal fare attività fisica
